# Associations between lipid-derived indices and cardiovascular–kidney–metabolic syndrome progression among Chinese middle-aged and elderly adults: a longitudinal study

**DOI:** 10.3389/fnut.2026.1742974

**Published:** 2026-03-25

**Authors:** Yu Tang, Wenyue Xu, Yuzhen Jiang, Chang Liu, Wenkai Zhu, Daoqin Liu, Xia Fu, Qiwen Wu

**Affiliations:** 1 Department of Laboratory, The First Affiliated Hospital of Wannan Medical College, Wuhu, Anhui, China; 2 Department of Nephrology, The First Affiliated Hospital of Wannan Medical College, Wuhu, Anhui, China

**Keywords:** cardiovascular disease risk, cardiovascular–kidney–metabolic syndrome, CHARLS, CKM progression, lipid-derived indices

## Abstract

**Background:**

The cardiovascular–kidney–metabolic syndrome (CKM) underscores the pathological interconnections between metabolic abnormalities, chronic kidney disease (CKD), and cardiovascular disease (CVD). Lipid metabolism is closely associated with the pathophysiology of these diseases. This study aimed to investigate the associations between eight lipid-derived indices and the progression of CKM syndrome.

**Methods:**

This study utilized data from the 2011–2020 China Health and Retirement Longitudinal Study (CHARLS). Lipid-derived indices were included: the atherogenic index of plasma (AIP), non-high-density lipoprotein cholesterol (Non-HDL-C), the non-HDL-C to HDL-C ratio (NHHR), the lipoprotein combined index (LCI), the remnant cholesterol (RC), the lipid accumulation product (LAP), the visceral adiposity index (VAI), and the triglyceride-glucose ratio (TyG). Cox regression analysis and restricted cubic spline (RCS) modeling were employed to assess the associations between lipid-derived indices and progression to CVD in the CKM syndrome stage 0–3 population. A comparison of prediction performance was conducted via the concordance index (C-index). Logistic regression and RCS models were used to analyze the associations between lipid-derived indices and the progression of CKM syndrome.

**Results:**

After adjusting for potential confounders, all lipid-derived indices, except for RC, exhibited positive associations with the risk of CVD in the CKM syndrome stages 0–3 population. The Cox regression model revealed that the AIP, non-HDL-C, NHHR, LCI, LAP, VAI, and TyG were positively associated with the risk of CVD. The RCS models demonstrated that the AIP, NHHR, LCI, and TyG were linearly associated with CVD risk, whereas the LAP and VAI exhibited nonlinear associations with CVD risk. The predictive model incorporating NHHR demonstrated the highest performance, with a C-index of 0.6322. Similarly, all lipid-derived indices, except RC, were positively correlated with the progression of CKM syndrome, as determined by logistic regression analysis.

**Conclusion:**

Seven lipid-derived indices—AIP, non-HDL-C, NHHR, LCI, LAP, VAI, and TyG—were positively associated with the risk of CVD in individuals diagnosed with CKM syndrome across stages 0–3. The NHHR demonstrated a stronger predictive value for CVD risk. Additionally, the seven indices were positively correlated with the progression of CKM syndrome, suggesting that these lipid-derived indices are important predictors of CKM progression.

## 1 Introduction

Cardiovascular diseases (e.g., ischemic heart disease, stroke), CKD, and metabolic disorders (e.g., diabetes) are leading causes of death globally and regionally, presenting a significant challenge to global healthcare (1). Numerous studies have confirmed the complex pathophysiologic links among the cardiovascular, renal, and metabolic systems (2, 3). The American Heart Association (AHA) has defined CKM syndrome and provided guidelines for its staging, prevention, and management (4). CKM syndrome is characterized by progressive development, often resulting from the accumulation of excess or dysfunctional adipose tissue (5).

Adipose tissue promotes the upregulation of proinflammatory adipokines and the downregulation of anti-inflammatory adipokines through various mechanisms, leading to a chronic inflammatory state (6). Chronic low-grade inflammation impairs insulin sensitivity, thereby promoting the progression of type 2 diabetes (7). Obesity is also linked to a range of metabolic abnormalities, including hypertension (8), dyslipidemia (9), and nonalcoholic fatty liver disease (NAFLD) (10). Over time, these factors interact to damage arterial vessels (11), the heart (12, 13), and kidney tissue (14). Additionally, metabolic abnormalities and impaired renal function may severely affect cardiovascular health, and these interacting factors significantly increase the risk of CVD (15). Overall, the staging of CKM syndrome divides the combined cardiovascular-renal-metabolic risk into distinct stages and reflects the syndrome’s dynamic progression.

Lipid abnormalities are key factors in the development and progression of CVD and CKD (16, 17). Various lipid-derived indices have been developed to assess lipid abnormalities and play critical roles in predicting various diseases. For example, non-HDL-C is a lipid-related index with strong predictive value in various diseases, particularly cardiovascular diseases (18). In addition to non-HDL-C, other metrics, such as the TyG, have demonstrated strong predictive power. The TyG index predicts cardiovascular and all-cause mortality in patients with CVD in diabetic or prediabetic populations (19). Furthermore, elevated TyG is strongly associated with the deterioration of renal function in elderly individuals (20). Overall, these findings highlight the potential role of lipid-derived indices in assessing health risk in patients with CKM syndrome, offering valuable insights for clinical decision-making.

This study aimed to assess the associations between lipid-derived indices and CVD in individuals with stage 0–3 CKM syndrome via data from the 2011–2020 CHARLS. Subgroup analyses were conducted to examine the influence of factors, such as age and sex, on these associations. This study focused on the relationship between lipid-related metrics and the staged progression of CKM syndrome, providing a more comprehensive understanding of CKM syndrome health risks in middle-aged and elderly populations. This approach offers valuable insights into the relationship between lipid-derived indices and CKM progression, contributing to improved risk prediction and public health strategies.

## 2 Methods

### 2.1 Study design and population

The CHARLS is a longitudinal survey of individuals aged 45 years and older in China that includes health, economic, and social data. The survey covered 28 provinces in China, with 150 county-level towns randomly selected via the probability proportional to size (PPS) sampling technique. The baseline survey was conducted in 2011, followed by follow-up surveys in 2013 (Wave 2), 2015 (Wave 3), 2018 (Wave 4), and 2020 (Wave 5) (21). CHARLS was approved by the Biomedical Ethics Review Committee of Peking University (IRB00001052-11015), and all participants provided written informed consent.

**Figure 1:**
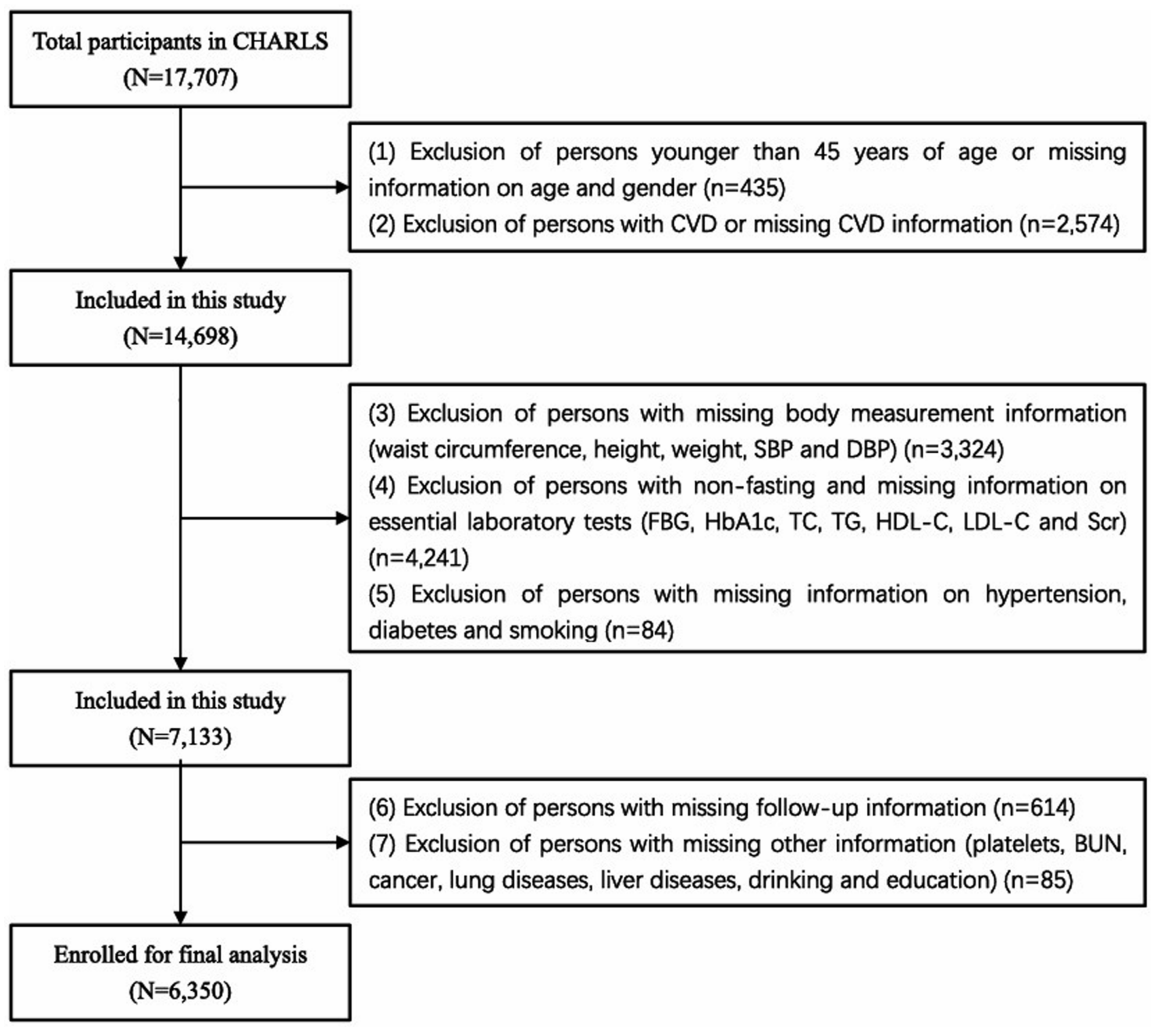
Flowchart of participant selection.

Our study used the 2011 baseline survey, which included a total of 17,707 participants. The flowchart (Figure 1) depicts the inclusion criteria for participants. (1) Individuals younger than 45 years or with missing information on age and sex were excluded. (2) Individuals with CVD or missing CVD data at baseline were excluded. (3) Individuals with missing body measurement data were excluded. (4) Individuals who underwent laboratory tests on a non-fasting basis or were missing the required laboratory tests were excluded. (5) Individuals with missing data on hypertension, diabetes, or smoking were excluded. (6) Individuals with missing follow-up data were excluded. (7) Individuals missing other data were excluded. Ultimately, a total of 6,350 participants were included in our study.

### 2.2 Definition of CKM syndrome stages 0–4

The definition of CKM syndrome staging was derived from the American Heart Association Presidential Advisory Statement (4): Stage 0: absence of CKM-related health risk factors; Stage 1: obesity, abdominal obesity, or prediabetes without other metabolic risks or CKD; Stage 2: metabolic issues (diabetes, hypertension, high triglycerides, and metabolic syndrome) or moderate to high-risk CKD; Stage 3: subclinical CVD, i.e., very high-risk CKD or high predicted 10-year risk of CVD; Stage 4: clinical CVD of CKM, including coronary heart disease, heart failure, stroke, peripheral arterial disease, and atrial fibrillation. The estimated glomerular filtration rate (eGFR) was calculated via the Chinese Modification of Diet in Renal Disease (C-MDRD) equation (22). The 10-year CVD risk was predicted via the Framingham CVD prediction score, with a Framingham risk score ≥ 21.5 for women and ≥21.6 for men indicating subclinical CVD (23).

### 2.3 Data collection

This study collects relevant data from the following datasets of CHARLS:

(1) Basic information: age, sex, education level and marital status.

(2) Health status and function: hypertension, hypertension medication, diabetes, diabetes medication, liver disease, lung disease, cancer, smoking and drinking status.

(3) Physical examination information: systolic blood pressure (SBP), diastolic blood pressure (DBP), height, weight and waist circumference.

(4) Blood test data: glycosylated hemoglobin (HbA1c), fasting blood glucose (FBG), triglyceride (TG), total cholesterol (TC), high-density lipoprotein cholesterol (HDL-c), low-density lipoprotein cholesterol (LDL-c), platelet, blood urea nitrogen (BUN), serum creatinine (Scr), C-reactive protein (CRP), and serum uric acid (SUA) levels.

Supplementary Table S1 describes the definitions of the variables in detail.

### 2.4 Definition of lipid-derived indices

The lipid-derived indices included TC, TG, HDL-C, LDL-C, WC and BMI. The specific formulas for the AIP, non-HDL-C, NHHR, LCI, RC, LAP, VAI and TyG are listed below:


$$\mathrm{BMI}=\text{weight}\;(\mathrm{kg})/{\text{height}}^{2}(m^{2})$$

AIP=lg(TG(mmol/L)/HDL−C(mmol/L))

Non−HDL−C=TC(mmol/L)–HDL−C(mmol/L)

$$\begin{array}{c} \text{NHHR}=(\mathrm{TC}\;(\text{mmol}/L)–\mathrm{HDL}-C\;(\text{mmol}/L))\\ /\mathrm{HDL}-C\;(\text{mmol}/L) \end{array}$$

$$\begin{array}{c} \mathrm{LCI}=\mathrm{TC}\;(\text{mmol}/L)\times \mathrm{TG}\;(\text{mmol}/L)\times \mathrm{LDL}-C\;(\text{mmol}/L)\\ /\mathrm{HDL}-C\;(\text{mmol}/L) \end{array}$$

RC=TC(mmol/L)–HDL−C(mmol/L)–LDL−C(mmol/L)

LAP(male)=(WC(cm)–65)×TG(mmol/L)

LAP(female)=(WC(cm)–58)×TG(mmol/L)

$$\begin{array}{c} \mathrm{VAI}\;(\text{male})=(\mathrm{WC}\;(\mathrm{cm})/(39.68+1.88\times \mathrm{BMI}\;(\mathrm{kg}/m^{2})))\\ \times (\mathrm{TG}\;(\text{mmol}/L)/1.03)\times (1.31/\mathrm{HDL}-C\;(\text{mmol}/L)) \end{array}$$

$$\begin{array}{c} \mathrm{VAI}\;(\text{female})=(\mathrm{WC}\;(\mathrm{cm})/(36.58+1.89\times \mathrm{BMI}\;(\mathrm{kg}/m^{2})))\\ \times (\mathrm{TG}\;(\text{mmol}/L)/0.81)\times (1.52/\mathrm{HDL}-C\;(\text{mmol}/L)) \end{array}$$

TyG=ln((TG(mg/dL)×FPG(mg/dL))/2)

### 2.5 Definition of outcome variables

In this study, the occurrence of a cardiovascular event (i.e., CKM stage 4) was used as the endpoint, with follow-up data collected from 2013 to 2020 (waves 2–4). In line with previous studies (24, 25), standardized questions were employed to gather information on the occurrence of cardiovascular events. Specifically, this was assessed via the following questions: “Have you ever been told by a doctor that you have had a myocardial infarction, coronary heart disease, angina pectoris, heart failure, or other heart conditions?” or “Have you ever been told by a doctor that you have had a stroke?” Participants who responded affirmatively were classified as having new-onset CVD. If, in a subsequent round of the validation questionnaire, a response from the previous round was contradicted, the response from the later questionnaire was considered definitive, and retrospective corrections were applied.

### 2.6 Statistical analysis

As none of the continuous variables followed a normal distribution (Supplementary Table S2), continuous variables are presented as medians with interquartile ranges (IQRs), and categorical variables are reported as frequencies (percentages). The Mann–Whitney *U* test or chi–square test was used for between-group comparisons. After excluding participants with missing essential data, the covariate with the most missing values was platelets, with a missing data rate of 0.793%. Consequently, participants with missing covariates were also excluded, and the missing covariates and their respective rates are presented in Supplementary Table S3. To control for potential confounders, a range of covariates were selected based on theoretical considerations and prior literature (26, 27). All covariates were measured at baseline.

Prior to analysis, non-HDL-C, NHHR, LCI, RC, LAP, and VAI were log-transformed (i.e., logarithmically transformed) to approximate a normal distribution. Cox hazards regression was employed to assess the associations between lipid-derived indices and CVD in the population with CKM syndrome stages 0–3. The proportional hazards assumption was tested separately for each Cox model using Schoenfeld residuals. Additionally, we examined the nonlinear relationship between lipid-derived indices and CVD risk via RCS, with knots placed at the 5th, 35th, 65th, and 95th percentiles of each exposure variable. To further investigate the relationships between lipid-derived indices and CVD risk in participants with CKM syndrome stages 0–3, subgroup and interaction analyses were conducted according to age, sex, hypertension, diabetes, and CKM syndrome stages. To evaluate the predictive value of lipid-derived indices for CVD risk, model performance was assessed via the C-index.

Additionally, we analyzed the associations between lipid-derived indices and the staged progression of CKM syndrome. As the CHARLS dataset included only blood test data from 2011 and 2015, CKM syndrome staging was performed for these 2 years. CKM syndrome staging was compared between the 2 years, with decreased or unchanged staging considered negative controls, and staging progression was defined as positive results. Logistic regression analysis was employed to assess the associations between lipid-derived indices and the staged progression of CKM syndrome.

To account for multiple testing across the eight lipid-related indices, we applied the Benjamini-Hochberg False Discovery Rate (FDR) correction with a *Q*-value threshold of 0.05. Associations with FDR-adjusted *p*-values (*q*-values) < 0.05 were considered statistically significant. To evaluate the robustness of the results, a sensitivity analysis was conducted. Median interpolation was applied to continuous variables, whereas missing values for categorical variables were categorized separately. The main analysis was then repeated after interpolation. All the statistical analyses were performed via R (version 4.3.3).

## 3 Results

### 3.1 Characteristics of the study population

A total of 6,350 participants, with a median age of 58 years (interquartile range: 52–65), were included in this study, 52.91% of whom were female. During the 9-year follow-up period, 1,453 participants developed CVD. The baseline characteristics of the study population, stratified by CVD status, are shown in Table 1. The participants with CVD exhibited significant differences in baseline characteristics (*p* < 0.05): a greater proportion of females, older age, and greater prevalence of non/previous smokers and alcohol drinkers. Additionally, there was a higher incidence of CKM syndrome stages 2–3, hypertension, diabetes mellitus, lung disease, and liver disease. Participants with CVD exhibited higher levels of BMI, WC, SBP, DBP, FPG, TG, TC, LDL-C, platelets, CRP, HbA1c, AIP, non-HDL-C, NHHR, LCI, RC, LAP, VAI, and TyG, while HDL-C and eGFR were lower.

**Table 1:** Baseline characteristics of subjects classified based on CVD and non-CVD in the CKM syndrome stages 0–3 population.

Variables	Total (*n* = 6,350)	Non-CVD (*n* = 4,897)	CVD (*n* = 1,453)	*p*-value
Age (year)	58.00 (52.00, 65.00)	58.00 (51.00, 65.00)	60.00 (54.00, 66.00)	<0.001
Sex, *n* (%)				0.016
Female	3,360 (52.91%)	2,551 (52.09%)	809 (55.68%)	
Male	2,990 (47.09%)	2,346 (47.91%)	644 (44.32%)	
Education level, *n* (%)				0.413
Below primary school	3,013 (47.45%)	2,312 (47.21%)	701 (48.25%)	
Primary school	1,442 (22.71%)	1,103 (22.52%)	339 (23.33%)	
Middle school	1,265 (19.92%)	998 (20.38%)	267 (18.38%)	
High school or above	630 (9.92%)	484 (9.88%)	146 (10.05%)	
Marital status, *n* (%)				0.946
Married	698 (10.99%)	539 (11.01%)	159 (10.94%)	
Others	5,652 (89.01%)	4,358 (88.99%)	1,294 (89.06%)	
Smoking status, *n* (%)				<0.001
Never	3,877 (61.06%)	2,980 (60.85%)	897 (61.73%)	
Former	517 (8.14%)	369 (7.54%)	148 (10.19%)	
Current	1956 (30.80%)	1,548 (31.61%)	408 (28.08%)	
Alcohol consumption, *n* (%)				0.003
Never	4,305 (67.80%)	3,286 (67.10%)	1,019 (70.13%)	
Former	375 (5.91%)	276 (5.64%)	99 (6.81%)	
Current	1,670 (26.30%)	1,335 (27.26%)	335 (23.06%)	
BMI (kg/m^2^)	23.09 (20.82, 25.70)	22.96 (20.71, 25.42)	23.73 (21.23, 26.57)	<0.001
WC (cm)	84.25 (77.60, 91.80)	84.00 (77.00, 91.00)	86.60 (79.20, 94.00)	<0.001
SBP (mmHg)	127.00 (114.33, 141.67)	126.00 (113.67, 140.33)	130.67 (117.33, 146.33)	<0.001
DBP (mmHg)	74.67 (67.33, 83.00)	74.33 (67.00, 82.33)	76.67 (68.33, 85.00)	<0.001
Hypertension, *n* (%)				<0.001
No	2,855 (44.96%)	2,345 (47.89%)	510 (35.10%)	
Yes	3,495 (55.04%)	2,552 (52.11%)	943 (64.90%)	
Diabetes, *n* (%)				0.001
No	5,394 (84.94%)	4,199 (85.75%)	1,195 (82.24%)	
Yes	956 (15.06%)	698 (14.25%)	258 (17.76%)	
Cancer, *n* (%)				0.416
No	6,310 (99.37%)	4,864 (99.33%)	1,446 (99.52%)	
Yes	40 (0.63%)	33 (0.67%)	7 (0.48%)	
Lung disease, *n* (%)				<0.001
No	5,754 (90.61%)	4,482 (91.53%)	1,272 (87.54%)	
Yes	596 (9.39%)	415 (8.47%)	181 (12.46%)	
Liver disease, *n* (%)				0.001
No	6,175 (97.24%)	4,780 (97.61%)	1,395 (96.01%)	
Yes	175 (2.76%)	117 (2.39%)	58 (3.99%)	
FPG (mg/dl)	102.06 (94.32, 112.14)	101.88 (94.32, 111.60)	102.78 (94.86, 113.40)	0.003
TG (mmol/l)	1.17 (0.84, 1.69)	1.15 (0.83, 1.68)	1.22 (0.89, 1.76)	<0.001
TC (mmol/l)	4.95 (4.35, 5.60)	4.93 (4.34, 5.57)	5.02 (4.40, 5.69)	0.002
HDL-C (mmol/l)	1.29 (1.06, 1.56)	1.30 (1.07, 1.57)	1.26 (1.04, 1.52)	<0.001
LDL-C (mmol/l)	2.98 (2.44, 3.58)	2.96 (2.43, 3.54)	3.05 (2.49, 3.66)	<0.001
Platelets (10^9^/L)	207.00 (162.00, 255.00)	206.00 (161.00, 254.00)	211.00 (167.00, 259.00)	0.022
Scr (mg/dl)	0.76 (0.64, 0.88)	0.76 (0.64, 0.88)	0.76 (0.66, 0.88)	0.843
BUN (mg/dl)	15.10 (12.52, 18.18)	15.15 (12.55, 18.26)	14.99 (12.44, 17.90)	0.101
CRP (mg/L)	1.01 (0.55, 2.09)	0.95 (0.53, 1.97)	1.20 (0.61, 2.32)	<0.001
SUA (mg/dl)	4.26 (3.55, 5.11)	4.27 (3.56, 5.12)	4.24 (3.51, 5.06)	0.229
HbA1c (%)	5.10 (4.90, 5.40)	5.10 (4.90, 5.40)	5.20 (4.90, 5.50)	<0.001
eGFR	118.95 (102.96, 136.65)	119.15 (103.35, 137.14)	117.61 (102.14, 135.38)	0.033
AIP	−0.05 (−0.24, 0.18)	−0.06 (−0.25, 0.17)	−0.01 (−0.22, 0.20)	<0.001
Non-HDL-C	3.60 (3.01, 4.27)	3.57 (2.99, 4.24)	3.72 (3.10, 4.35)	<0.001
NHHR	2.81 (2.10, 3.74)	2.77 (2.05, 3.69)	2.96 (2.20, 3.92)	<0.001
LCI	13.40 (7.41, 25.38)	12.89 (7.21, 24.26)	15.28 (8.20, 28.66)	<0.001
RC	0.50 (0.30, 0.81)	0.50 (0.29, 0.80)	0.53 (0.33, 0.84)	0.003
LAP	26.06 (14.57, 46.04)	25.00 (13.93, 44.42)	30.66 (17.43, 51.00)	<0.001
VAI	1.44 (0.88, 2.50)	1.39 (0.85, 2.45)	1.59 (1.00, 2.67)	<0.001
TyG	8.58 (8.22, 9.01)	8.56 (8.20, 9.00)	8.64 (8.29, 9.06)	<0.001
CKM stage, *n* (%)				<0.001
0	562 (8.85%)	487 (9.94%)	75 (5.16%)	
1	1,119 (17.62%)	910 (18.58%)	209 (14.38%)	
2	2,786 (43.87%)	2,132 (43.54%)	654 (45.01%)	
3	1883 (29.65%)	1,368 (27.94%)	515 (35.44%)	

BMI, body mass index; WC, waist circumference; SBP, systolic blood pressure; DBP, diastolic blood pressure; FPG, fasting plasma glucose; TG, triglyceride; TC, total cholesterol; HDL-C, high-density lipoprotein cholesterol; LDL-C, low-density lipoprotein cholesterol; Scr, serum creatinine; BUN, blood urea nitrogen; CRP, C-reactive protein; SUA, serum uric acid; HbA1c, glycosylated hemoglobin A1c; eGDR, estimated glucose disposal rate; AIP, atherogenic index of plasma; Non-HDL-C, Non-high-density lipoprotein cholesterol; NHHR, Non-HDL-C to HDL-C ratio; LCI, lipoprotein combined index; RC, remnant cholesterol; LAP, lipid accumulation product; VAI, visceral adiposity index; TyG, triglyceride-glucose. The continuous variables are expressed as medians (interquartile spacing). Categorical variables are presented as numbers (percentages).

Continuous variables are expressed as medians (interquartile range [IQR]). Categorical variables are expressed as numbers (percentages).

### 3.2 Relationship between lipid-derived indices and CVD incidence in a population with CKM syndrome stages 0–3

The associations between lipid-derived indices and CVD risk in the CKM syndrome stages 0–3 population are presented in Table 2. Except for RC, all other lipid-derived indices were significantly positively associated with CVD risk (*p* < 0.05). The association remained statistically significant after multivariable adjustment and FDR correction. In Model 3, the hazard ratios (HR) and 95% confidence intervals (95% CI) for the lipid-derived indices were as follows: AIP (HR = 1.26, 95% CI: 1.06–1.49), non-HDL-C (HR = 1.82, 95% CI: 1.14–2.92), NHHR (HR = 1.65, 95% CI: 1.23–2.21), LCI (HR = 1.29, 95% CI: 1.12–1.49), RC (HR = 1.02, 95% CI: 0.91–1.15), LAP (HR = 1.35, 95% CI: 1.17–1.55), VAI (HR = 1.28, 95% CI: 1.09–1.50), and TyG (HR = 1.12, 95% CI: 1.02–1.22). The proportional hazards assumption was tested separately for each Cox model using Schoenfeld residuals. As shown in Supplementary Table S4, all eight lipid-derived indices satisfied the assumption (all global *p* > 0.05).

**Table 2:** Associations between lipid-derived indices and CVD incidence in a population with CKM syndrome stages 0–3.

Variables	Model 1		Model 2		Model 3		FDR-q
	HR (95%CI)	*P*-value	HR (95%CI)	*P*-value	HR (95%CI)	*P*-value	
AIP	1.39 (1.19, 1.62)	<0.001	1.41 (1.21, 1.65)	<0.001	1.26 (1.06, 1.49)	0.007	0.011
Non-HDL-C	2.68 (1.71, 4.21)	<0.001	2.40 (1.52, 3.80)	<0.001	1.82 (1.14, 2.92)	0.013	0.017
NHHR	2.06 (1.56, 2.71)	<0.001	2.00 (1.51, 2.65)	<0.001	1.65 (1.23, 2.21)	<0.001	0.002
LCI	1.46 (1.27, 1.67)	<0.001	1.43 (1.25, 1.64)	<0.001	1.29 (1.12, 1.49)	<0.001	0.002
RC	1.10 (0.97, 1.25)	0.150	1.11 (0.98, 1.27)	0.104	1.02 (0.91, 1.15)	0.720	0.720
LAP	1.49 (1.31, 1.70)	<0.001	1.51 (1.32, 1.73)	<0.001	1.35 (1.17, 1.55)	<0.001	<0.001
VAI	1.43 (1.24, 1.65)	<0.001	1.43 (1.23, 1.66)	<0.001	1.28 (1.09, 1.50)	0.002	0.004
TyG	1.20 (1.11, 1.29)	<0.001	1.20 (1.11, 1.30)	<0.001	1.12 (1.02, 1.22)	0.019	0.022

Model 1: unadjusted.

Model 2: adjusted for sex, age, smoking status, alcohol consumption, education level, and marital status.

Model 3: adjusted for Model 2 covariates plus hypertension, diabetes, cancer, lung disease, liver disease, eGFR, platelets, BUN, CRP, and SUA.

HR, hazard ratio; CI, confidence interval; FDR, false discovery rate.

**Figure 2:**
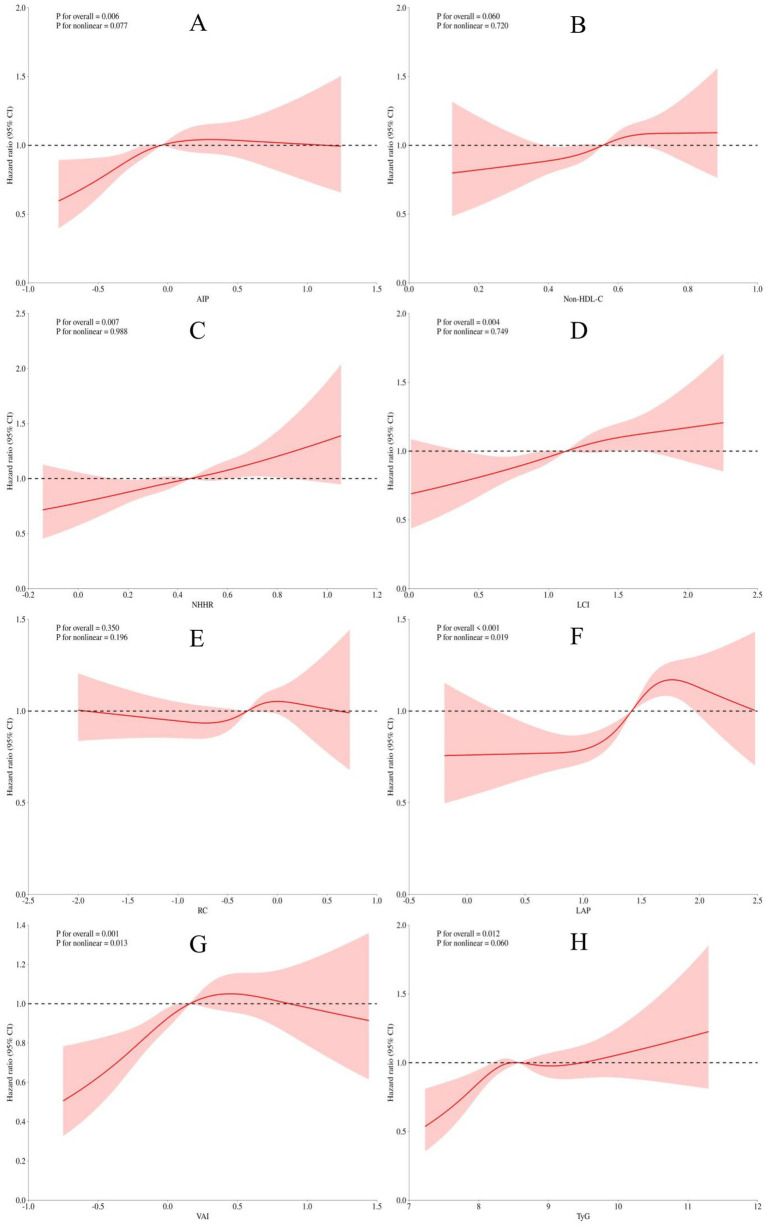
Restricted cubic spline analysis of lipid-derived indices (**A**: AIP; **B**: Non-HDL-C; **C**: NHHR; **D**: LCI; **E**: RC; **F**: LAP; **G**: VAI; **H**: TyG) in relation to the risk of CVD incidence in a population with CKM syndrome stages 0–3. The model was adjusted for sex, age, smoking status, alcohol consumption, education level, marital status, hypertension, diabetes, cancer, lung disease, liver disease, eGFR, platelet count, BUN, CRP, and SUA.

Additionally, we employed RCS to analyze the nonlinear associations between eight lipid-derived indices and CVD risk. As shown in Figure 2, after adjusting for confounders, the AIP, NHHR, LCI, and TyG were linearly associated with CVD risk (*P* for overall < 0.05, *P* for non-linearity > 0.05; Figures 2A,C,D,H), whereas the LAP and VAI were non-linearly associated with CVD risk (*P* for non-linearity < 0.05; Figures 2F,G).

To further investigate the associations between lipid-derived indices and CVD risk in the CKM syndrome stages 0–3 population, subgroup analyses were conducted on the basis of age, sex, hypertension, diabetes, and CKM syndrome stage. As shown in Table 3 and Supplementary Table S5, no significant interactions were detected in any of the subgroups analyzed (*P* for interaction > 0.05).

**Table 3:** Subgroup analyses of associations between lipid-derived indices and CVD incidence in a population with CKM syndrome stages 0–3.

Variables	Number of participants	HR (95%CI)	*P*-value	*P* for interaction
AIP				
Sex				0.143
Female	3,360	1.08 (0.85, 1.36)	0.542	
Male	2,990	1.55 (1.21, 1.99)	<0.001	
Age (years)				0.990
< 60	3,551	1.19 (0.95, 1.50)	0.136	
≥ 60	2,799	1.33 (1.04, 1.70)	0.021	
Non-HDL-C				
Sex				0.324
Female	3,360	1.46 (0.76, 2.83)	0.255	
Male	2,990	2.44 (1.22, 4.88)	0.012	
Age (years)				0.668
< 60	3,551	1.44 (0.74, 2.82)	0.284	
≥ 60	2,799	2.43 (1.24, 4.77)	0.010	
NHHR				
Sex				0.172
Female	3,360	1.28 (0.85, 1.94)	0.240	
Male	2,990	2.26 (1.47, 3.47)	<0.001	
Age (years)				0.921
< 60	3,551	1.56 (1.03, 2.37)	0.036	
≥ 60	2,799	1.75 (1.15, 2.65)	0.009	
LCI				
Sex				0.131
Female	3,360	1.14 (0.93, 1.39)	0.210	
Male	2,990	1.52 (1.23, 1.88)	<0.001	
Age (years)				0.751
< 60	3,551	1.19 (0.97, 1.46)	0.095	
≥ 60	2,799	1.42 (1.15, 1.74)	0.001	
RC				
Sex				0.963
Female	3,360	1.01 (0.87, 1.17)	0.920	
Male	2,990	1.04 (0.86, 1.26)	0.655	
Age (years)				0.083
< 60	3,551	1.16 (0.94, 1.44)	0.176	
≥ 60	2,799	0.99 (0.88, 1.12)	0.846	
LAP				
Sex				0.785
Female	3,360	1.28 (1.04, 1.58)	0.019	
Male	2,990	1.42 (1.16, 1.74)	<0.001	
Age (years)				0.687
< 60	3,551	1.26 (1.02, 1.55)	0.028	
≥ 60	2,799	1.42 (1.16, 1.74)	<0.001	
VAI				
Sex				0.208
Female	3,360	1.13 (0.91, 1.40)	0.277	
Male	2,990	1.53 (1.21, 1.94)	<0.001	
Age (years)				0.896
< 60	3,551	1.19 (0.95, 1.49)	0.125	
≥ 60	2,799	1.38 (1.10, 1.74)	0.005	
TyG				
Sex				0.826
Female	3,360	1.06 (0.94, 1.20)	0.358	
Male	2,990	1.20 (1.05, 1.38)	0.007	
Age (years)				0.979
< 60	3,551	1.08 (0.95, 1.22)	0.250	
≥ 60	2,799	1.17 (1.02, 1.34)	0.023	

The model was adjusted for sex, age, smoking status, alcohol consumption, education level, marital status, hypertension, diabetes, cancer, lung disease, liver disease, eGFR, platelet count, BUN, CRP, and SUA (excluding the stratification variable).

Table 4 presents the results of the performance comparison of the eight prediction models, with C-index values ranging from 0.6256 to 0.6322. The NHHR model exhibited the best performance, with a C-index of 0.6322 (95% CI: 0.6055–0.6589). Compared with the TyG model, the NHHR model significantly improved the prediction of CVD (*p* = 0.045).

**Table 4:** Comparison of CVD prediction model performance.

Model	C-index	95% CI	ΔC (95%CI)	*P*–value
TyG	0.6256	(0.5988, 0.6524)	Ref	
VAI	0.6268	(0.5999, 0.6536)	0.0012 (−0.0019, 0.0047)	0.488
AIP	0.6278	(0.6009, 0.6546)	0.0021 (0.0000, 0.0048)	0.078
RC	0.6287	(0.6021, 0.6554)	0.0031 (−0.0023, 0.0077)	0.230
LAP	0.6301	(0.6032, 0.6570)	0.0045 (−0.0020, 0.0110)	0.173
LCI	0.6302	(0.6036, 0.6568)	0.0046 (−0.0011, 0.0101)	0.111
NHDL	0.6314	(0.6049, 0.6579)	0.0058 (−0.0020, 0.0121)	0.109
NHHR	0.6322	(0.6055, 0.6589)	0.0066 (0.0000, 0.0128)	0.045

The model was adjusted for sex, age, smoking status, alcohol consumption, education level, marital status, hypertension, diabetes, cancer, lung disease, liver disease, eGFR, platelet count, BUN, CRP, and SUA. C-index, concordance index; CI, confidence interval; ΔC, change in the concordance index.

### 3.3 Relationship between lipid-derived indices and progression of CKM syndrome

Table 5 presents the relationships between lipid-derived indices and the progression of CKM syndrome. In Model 1, the AIP, non-HDL-C, NHHR, LCI, RC, LAP, VAI, and TyG were negatively associated with the progression of CKM syndrome staging. In contrast, in Models 2 and 3, AIP, non-HDL-C, NHHR, LCI, LAP, VAI, and TyG were positively associated with CKM syndrome stage progression (*p* < 0.05). After FDR correction for multiple testing in Model 3, these associations retained statistical significance. However, RC showed no significant association with CKM syndrome stage progression (*p* > 0.05). Supplementary Table S6 shows that when not adjusted for baseline CKM stage, the AIP, non-HDL-C, NHHR, LCI, RC, LAP, VAI, and TyG were negatively associated with CKM syndrome stage progression in all the models. Supplementary Table S7 shows that the AIP, non-HDL-C, NHHR, LCI, RC, LAP, VAI, and TyG were the positively associated with baseline CKM syndrome stage in all the models.

**Table 5:** Associations of lipid-related indices with stage progression of CKM syndrome.

Variables	Model 1		Model 2		Model 3		FDR-q
	OR (95%CI)	*P*-value	OR (95%CI)	*P*-value	OR (95%CI)	*P*-value	
AIP	0.32 (0.25, 0.39)	<0.001	1.54 (1.17, 2.02)	0.002	1.73 (1.30, 2.30)	<0.001	<0.001
Non-HDL-C	0.16 (0.09, 0.28)	<0.001	2.55 (1.25, 5.17)	0.010	2.81 (1.35, 5.84)	0.006	<0.007
NHHR	0.22 (0.15, 0.31)	<0.001	2.64 (1.66, 4.19)	<0.001	3.08 (1.91, 4.97)	<0.001	<0.001
LCI	0.41 (0.35, 0.49)	<0.001	1.54 (1.22, 1.94)	<0.001	1.68 (1.32, 2.14)	<0.001	<0.001
RC	0.58 (0.49, 0.68)	<0.001	1.08 (0.92, 1.26)	0.368	1.10 (0.93, 1.29)	0.274	0.274
LAP	0.51 (0.44, 0.60)	<0.001	1.89 (1.51, 2.36)	<0.001	1.94 (1.54, 2.44)	<0.001	0.001
VAI	0.39 (0.32, 0.48)	<0.001	1.53 (1.18, 1.97)	0.001	1.68 (1.29, 2.20)	<0.001	0.001
TyG	0.49 (0.44, 0.55)	<0.001	1.24 (1.08, 1.43)	0.003	1.27 (1.09, 1.48)	0.002	0.003

Model 1: unadjusted.

Model 2: adjusted for sex, age, smoking status, alcohol consumption, education level, marital status, and CKM.

Model 3: adjusted for Model 2 covariates plus hypertension, diabetes, cancer, lung disease, liver disease, eGFR, platelets, BUN, CRP, and SUA.

OR, odds ratio; CI, confidence interval; FDR, false discovery rate.

Additionally, we employed RCS to analyze the nonlinear relationships between eight lipid-derived indices and CKM syndrome stage progression. As shown in Supplementary Figure S1, after adjusting for confounders, AIP, NHHR, LCI, and TyG were linearly and positively associated with CKM syndrome stage progression (*P* for overall < 0.05, *P* for non-linear > 0.05; Supplementary Figures S1A,C,D,H), whereas non-HDL-C, LAP, and VAI were nonlinearly associated with CKM syndrome stage progression (*P* for non-linear < 0.05; Supplementary Figures S1B,F,G).

### 3.4 Sensitivity analysis

After imputing missing covariates, the associations between lipid-derived indices and CVD incidence in the CKM syndrome stages 0–3 population were reanalyzed (Supplementary Table S8), as were the associations between lipid-derived indices and the progression of CKM syndrome stages (Supplementary Table S9). Compared with the analysis prior to covariate imputation, the results remained consistent with the main analysis.

## 4 Discussion

In this analysis, using data from the CHARLS cohort spanning 2011–2020, we examined the associations between eight lipid-derived indices and the risk of CVD in individuals with CKM syndrome stages 0–3. Our findings suggest that, with the exception of RC, other lipid-related markers, including the AIP, non-HDL-C, NHHR, LCI, LAP, VAI, and TyG, are positively associated with the risk of CVD in individuals with CKM syndrome stages 0–3. Notably, no significant interactions were observed for any lipid-derived indices in subgroup analyses based on age, sex, hypertension, diabetes mellitus, and CKM syndrome stages 0–3. These results indicate that the associations between lipid-related markers and CVD risk remain stable across these subgroups. C-statistic analysis revealed that the NHHR model was more predictive of CVD risk in the CKM syndrome stage 0–3 population. Furthermore, lipid-derived indices, with the exception of RC, were positively associated with CKM syndrome progression. These findings underscore the potential predictive value of these lipid-derived indices for risk progression in CKM syndrome patients.

Abnormalities in lipid metabolism are key drivers of atherosclerosis (28, 29), the primary pathological basis of most cardiovascular diseases (30, 31). Among the various lipid components, high-density lipoprotein (HDL) is thought to possess several antiatherosclerotic properties, exerting cardiovascular protective effects through reverse cholesterol transport as well as antioxidant and anti-inflammatory actions (32, 33). In contrast, high triglyceride levels also play a significant role in the development of atherosclerosis and have emerged as prevalent risk factors for CVD (34, 35). Additionally, oxidative modification of low-density lipoprotein (LDL) constitutes a critical pathogenic mechanism in atherogenesis (36, 37). Furthermore, total cholesterol is positively associated with ischemic heart disease mortality in middle-aged and older adults (38). Given the central role of lipid metabolism in cardiovascular pathophysiology, lipid-derived indices may serve as effective predictors of the development and progression of CKM syndrome.

Non-HDL-C, which encompasses LDL-C, medium-density lipoprotein (MDL), and very low-density lipoprotein (VLDL) remnants, is more strongly associated with the risk of cardiovascular events than LDL-C alone (39, 40). The NHHR is defined as the ratio of non-HDL-C to HDL-C. Compared with either of its components alone, the NHHR has demonstrated superior predictive ability for cardiovascular events (41–43). This enhanced predictive ability may be attributed to its comprehensive nature, integrating information on both atherogenic and anti-atherogenic lipids (44). In this study, we observed that the NHHR outperformed other indices in predicting CVD risk in individuals with CKM syndrome stages 0–3.

This study focused on the relationship between lipid-derived indices and the staged progression of CKM syndrome. The findings of this study align with those of previous studies. For instance, Hu et al. (45) reported a significant correlation between AIP levels and CVD risk, with no significant interaction observed in subgroup analyses. Our findings concerning the TyG index are consistent with those of a study conducted in a Chinese population, which reported a positive correlation between the TyG index and the progression of CKM syndrome (46). Previous studies have shown that abnormalities in lipid metabolism play crucial roles in the development and progression of CVD, diabetes mellitus, and CKD (47–50). Abnormal lipid metabolism, as a key influence on CKM syndrome, affects all stages of the condition and significantly contributes to its disease burden.

In this study, we observed that the association between lipid-derived indices and the staged progression of CKM syndrome shifted from negative to positive after adjusting for baseline CKM. Lipid-related indices were positively correlated with the baseline CKM stage. These findings suggest that baseline CKM is a critical confounder in the relationship between lipid-derived indices and CKM syndrome progression. One possible explanation for this phenomenon is that patients with a high lipid-derived index have a high baseline CKM stage and have limited scope for further deterioration, and therefore are less likely to deteriorate in CKM syndrome staging. In contrast, patients with a low baseline CKM stage have more room for deterioration and a greater likelihood of progression. However, this hypothesis needs to be further tested in subsequent studies.

However, this study has several limitations. First, the CKM staging system was adapted from American Heart Association guidelines and has not been formally validated in Chinese populations or for CHARLS-specific measurements. Although the core components of CKM syndrome are internationally recognized, ethnic differences in metabolic profiles may affect the applicability of this staging framework. Furthermore, due to the nature of CHARLS data, our assessment of CKM components—such as CKD risk and subclinical CVD—relied on surrogate metrics rather than gold-standard diagnoses, which may introduce bias. Second, although we adjusted for known major confounders, the potential influence of unmeasured factors cannot be excluded. Third, disease-related information was obtained from participants’ self-reports, which may have led to inaccuracies in estimating disease prevalence. Fourth, while NHHR demonstrated a higher predictive value compared with triglycerides alone, the incremental improvement was limited and of borderline statistical significance. This suggests that NHHR may not offer a substantial clinical advantage in predicting CKM progression, though it could serve as a complementary risk indicator or be more useful in specific subgroups—an issue that warrants further investigation. Finally, this study was conducted exclusively among Chinese adults aged ≥45 years in the CHARLS cohort. Therefore, the findings may not be generalizable to other ethnicities or healthcare systems with different disease prevalence and management practices. Future studies are needed to validate our findings in more diverse populations and clinical settings, and to determine whether population-specific modifications to the CKM staging criteria enhance its predictive utility for CKM syndrome progression.

## 5 Conclusion

In summary, seven lipid-derived indices—AIP, non-HDL-C, NHHR, LCI, LAP, VAI, and TyG—were positively associated with the risk of CVD in individuals diagnosed with CKM syndrome across stages 0–3. The NHHR has the highest predictive value for CVD risk, but further validation and evaluation of its clinical utility is needed. Additionally, the seven indices were positively correlated with the progression of CKM syndrome stage, highlighting their potential as important predictors of CKM progression. Future studies should further investigate and validate these findings to establish a basis for early prediction and intervention in CKM syndrome patients.

## Data Availability

Publicly available datasets were analyzed in this study. This data can be found at: https://charls.pku.edu.cn.
