# Supplementary material for: Associations between lipid-derived indices and cardiovascular–kidney–metabolic syndrome progression among Chinese middle-aged and elderly adults: a longitudinal study
Source: Front Nutr. 2026 Mar 25;13:1742974. doi: 10.3389/fnut.2026.1742974 (PMC13057432; doi:10.3389/fnut.2026.1742974)
Supplement: Supplementary file 1 [file Supplementary_file_1.DOCX]

**Table S1. Specific definitions of variables**

| Education level | 1. Below primary school (uneducated or incomplete primary education) 2. Primary school (private/home school or elementary school graduation) 3. Middle school 4. High school or above (high school, vocational school, college degree, bachelor's degree, master's degree, and doctoral degree) |
| --- | --- |
| Marital status | 1. Married (including temporary separation due to work) 2. Others (separated, divorced, widowed, and unmarried) |
| Smoking status | Smoking more than 100 cigarettes in a lifetime was defined as smoking.   1. Never 2. Former (who have quit) 3. Current (who continue to smoke) |
| Alcohol consumption | 1. Never (individuals who never or rarely drink alcohol or who drink less than once a month) 2. Former (individuals who drank more than once a month but less than once a month in the past year) 3. Current (individuals who drink more than once a month) |
| Overweight | BMI ≥23 kg/m2 |
| Abdominal obesity | Waist circumference≥80/90 cm in female/male |
| Hypertriglyceridemia | Triglycerides ≥ 135 mg/dL |
| Hypertension | SBP ≥130 mm Hg or DBP ≥80 mm Hg or self-reported diagnosis of hypertension or use of antihypertensive medications |
| Diabetes | Fasting blood glucose ≥ 125 mg/dL or HbA1c ≥ 6.5% or self-reported diagnosis of diabetes, use of insulin, or oral hypoglycemic agents |
| Prediabetes | Fasting blood glucose ≥ 100-124 mg/dL or HbA1c ≥ 5.7%-6.4% and  without self-reported diagnosis of diabetes, use of insulin, or oral  hypoglycemic agents |
| Prediabetes | Fasting blood glucose ≥ 100-124 mg/dL or HbA1c ≥ 5.7%-6.4% and without self-reported diagnosis of diabetes, use of insulin, or oral  hypoglycemic agents |
| Estimated glomerular filtration rate | 1. 30–60 mL/min/1.73 m² indicates an intermediate to high risk of CKD 2. eGFR <30 mL/min/1.73 m² indicates a very high risk of CKD |
| Metabolic syndrome | Meet any three or more of the five  ① Waist circumference ≥ 80/90 cm in female/male.  ② HDL cholesterol <50/40 mg/dL in female/male.  ③ Triglycerides>150 mg/dL or current use of lipid-lowering medications.  ④ Elevated blood pressure (SBP>130 mm Hg or DBP ≥80 mm Hg and/or use of antihypertensive medications) or current use of antihypertensive medications  ⑤ Fasting blood glucose >100 mg/dL or self-reported history of diabetes or current use of antidiabetic medications |

**TableS2. Test of normality**

| **Kolmogorov-Smirnov** | **Statistic** | **p value** |
| --- | --- | --- |
| AIP | 0.042 | <0.001 |
| Non-HDL-C | 0.046 | <0.001 |
| NHHR | 0.084 | <0.001 |
| LCI | 0.186 | <0.001 |
| RC | 0.157 | <0.001 |
| LAP | 0.161 | <0.001 |
| VAI | 0.243 | <0.001 |
| TyG | 0.058 | <0.001 |
| Age | 0.064 | <0.001 |
| BMI | 0.046 | <0.001 |
| WC | 0.061 | <0.001 |
| SBP | 0.064 | <0.001 |
| DBP | 0.040 | <0.001 |
| eGFR | 0.066 | <0.001 |
| FPG | 0.229 | <0.001 |
| TG | 0.160 | <0.001 |
| TC | 0.040 | <0.001 |
| HDL-C | 0.056 | <0.001 |
| LDL-C | 0.036 | <0.001 |
| Platelets | 0.044 | <0.001 |
| Scr | 0.096 | <0.001 |
| BUN | 0.067 | <0.001 |
| CRP | 0.365 | <0.001 |
| UA | 0.053 | <0.001 |
| HbA1c | 0.213 | <0.001 |

**Table S3. Distribution of variables with missing data**

| **Variables** | **Number of Missing** | **Missing proportion** |
| --- | --- | --- |
| Platelets | 51 | 0.793% |
| BUN | 1 | 0.016% |
| Cancer | 11 | 0.171% |
| Lung disease | 5 | 0.078% |
| Liver disease | 17 | 0.264% |
| Drinking status | 4 | 0.062% |
| Education level | 1 | 0.016% |
|  |  |  |

**Table S4. Testing the proportional hazards assumption for Cox models**

| **Lipid index** | **Global χ²** | **df** | **P value** |
| --- | --- | --- | --- |
| AIP | 12.806 | 17.00 | 0.749 |
| Non-HDL-C | 15.720 | 17.00 | 0.544 |
| NHHR | 14.419 | 17.00 | 0.637 |
| LCI | 13.699 | 17.00 | 0.688 |
| RC | 12.969 | 17.00 | 0.738 |
| LAP | 12.673 | 17.00 | 0.758 |
| VAI | 12.883 | 17.00 | 0.744 |
| TyG | 12.815 | 17.00 | 0.749 |

Note: P values were derived from the global test of Schoenfeld residuals. A P value > 0.05 indicates that the proportional hazards assumption is satisfied.

**Table S5. Subgroup analyses of associations between lipid-related indices and CVD incidence in a population with CKM syndrome stages 0-3**

| **Variables** | **Number of participants** | **HR (95%CI)** | **P value** | **P for interaction** |
| --- | --- | --- | --- | --- |
| **AIP** |  |  |  |  |
| Hypertension |  |  |  | 0.811 |
| No | 2855 | 1.29 (0.96, 1.74) | 0.088 |  |
| Yes | 3495 | 1.21 (0.99, 1.49) | 0.064 |  |
| Diabetes |  |  |  | 0.632 |
| No | 5394 | 1.32 (1.09, 1.61) | 0.005 |  |
| Yes | 956 | 1.05 (0.75, 1.48) | 0.781 |  |
| CKM stage |  |  |  | 0.930 |
| 0 | 562 | 1.27 (0.36, 4.55) | 0.710 |  |
| 1 | 1119 | 1.49 (0.70, 3.17) | 0.303 |  |
| 2 | 2786 | 1.27 (0.97, 1.65) | 0.080 |  |
| 3 | 1883 | 1.10 (0.84, 1.46) | 0.487 |  |
| **Non-HDL-C** |  |  |  |  |
| Hypertension |  |  |  | 0.299 |
| No | 2855 | 2.37 (1.06, 5.30) | 0.036 |  |
| Yes | 3495 | 1.49 (0.83, 2.67) | 0.180 |  |
| Diabetes |  |  |  | 0.979 |
| No | 5394 | 1.94 (1.15, 3.29) | 0.013 |  |
| Yes | 956 | 1.03 (0.34, 3.08) | 0.964 |  |
| CKM stage |  |  |  | 0.886 |
| 0 | 562 | 1.90 (0.17, 21.01) | 0.599 |  |
| 1 | 1119 | 1.48 (0.39, 5.60) | 0.560 |  |
| 2 | 2786 | 1.41 (0.68, 2.95) | 0.355 |  |
| 3 | 1883 | 1.67 (0.73, 3.80) | 0.221 |  |
| **NHHR** |  |  |  |  |
| Hypertension |  |  |  | 0.932 |
| No | 2855 | 1.81 (1.09, 3.00) | 0.022 |  |
| Yes | 3495 | 1.50 (1.04, 2.15) | 0.030 |  |
| Diabetes |  |  |  | 0.996 |
| No | 5394 | 1.73 (1.24, 2.41) | 0.001 |  |
| Yes | 956 | 1.22 (0.63, 2.35) | 0.552 |  |
| CKM stage |  |  |  | 0.842 |
| 0 | 562 | 1.20 (0.21, 6.70) | 0.837 |  |
| 1 | 1119 | 1.13 (0.44, 2.93) | 0.799 |  |
| 2 | 2786 | 1.71 (1.06, 2.76) | 0.027 |  |
| 3 | 1883 | 1.40 (0.84, 2.34) | 0.191 |  |
| **LCI** |  |  |  |  |
| Hypertension |  |  |  | 0.620 |
| No | 2855 | 1.37 (1.06, 1.76) | 0.015 |  |
| Yes | 3495 | 1.23 (1.02, 1.47) | 0.026 |  |
| Diabetes |  |  |  | 0.976 |
| No | 5394 | 1.32 (1.12, 1.56) | <0.001 |  |
| Yes | 956 | 1.06 (0.76, 1.49) | 0.734 |  |
| CKM stage |  |  |  | 0.958 |
| 0 | 562 | 1.43 (0.56, 3.62) | 0.450 |  |
| 1 | 1119 | 1.32 (0.78, 2.21) | 0.300 |  |
| 2 | 2786 | 1.22 (0.97, 1.54) | 0.091 |  |
| 3 | 1883 | 1.24 (0.96, 1.60) | 0.096 |  |
| **RC** |  |  |  |  |
| Hypertension |  |  |  | 0.345 |
| No | 2855 | 1.15 (0.91, 1.46) | 0.227 |  |
| Yes | 3495 | 0.96 (0.84, 1.10) | 0.577 |  |
| Diabetes |  |  |  | 0.673 |
| No | 5394 | 1.02 (0.90, 1.16) | 0.764 |  |
| Yes | 956 | 1.01 (0.72, 1.41) | 0.974 |  |
| CKM stage |  |  |  | 0.108 |
| 0 | 562 | 0.66 (0.46, 0.94) | 0.022 |  |
| 1 | 1119 | 1.29 (0.83, 2.01) | 0.262 |  |
| 2 | 2786 | 1.12 (0.89, 1.40) | 0.340 |  |
| 3 | 1883 | 0.91 (0.80, 1.04) | 0.188 |  |
| **LAP** |  |  |  |  |
| Hypertension |  |  |  | 0.637 |
| No | 2855 | 1.28 (1.01, 1.61) | 0.038 |  |
| Yes | 3495 | 1.37 (1.14, 1.64) | <0.001 |  |
| Diabetes |  |  |  | 0.336 |
| No | 5394 | 1.35 (1.15, 1.58) | <0.001 |  |
| Yes | 956 | 1.31 (0.94, 1.83) | 0.111 |  |
| CKM stage |  |  |  | 0.708 |
| 0 | 562 | 1.43 (0.67, 3.04) | 0.357 |  |
| 1 | 1119 | 1.27 (0.81, 2.01) | 0.301 |  |
| 2 | 2786 | 1.42 (1.12, 1.80) | 0.004 |  |
| 3 | 1883 | 1.18 (0.93, 1.49) | 0.177 |  |
| **VAI** |  |  |  |  |
| Hypertension |  |  |  | 0.821 |
| No | 2855 | 1.30 (0.99, 1.72) | 0.061 |  |
| Yes | 3495 | 1.24 (1.02, 1.51) | 0.028 |  |
| Diabetes |  |  |  | 0.924 |
| No | 5394 | 1.34 (1.11, 1.61) | 0.002 |  |
| Yes | 956 | 1.08 (0.78, 1.50) | 0.623 |  |
| CKM stage |  |  |  | 0.776 |
| 0 | 562 | 1.39 (0.43, 4.50) | 0.578 |  |
| 1 | 1119 | 1.58 (0.80, 3.12) | 0.183 |  |
| 2 | 2786 | 1.29 (1.00, 1.66) | 0.046 |  |
| 3 | 1883 | 1.12 (0.86, 1.45) | 0.409 |  |
| **TyG** |  |  |  |  |
| Hypertension |  |  |  | 0.988 |
| No | 2855 | 1.16 (0.98, 1.36) | 0.079 |  |
| Yes | 3495 | 1.08 (0.97, 1.21) | 0.148 |  |
| Diabetes |  |  |  | 0.608 |
| No | 5394 | 1.15 (1.03, 1.28) | 0.013 |  |
| Yes | 956 | 1.02 (0.86, 1.21) | 0.789 |  |
| CKM stage |  |  |  | 0.408 |
| 0 | 562 | 1.22 (0.60, 2.48) | 0.588 |  |
| 1 | 1119 | 1.49 (0.96, 2.31) | 0.079 |  |
| 2 | 2786 | 1.10 (0.95, 1.26) | 0.203 |  |
| 3 | 1883 | 1.03 (0.89, 1.20) | 0.683 |  |
|  |  |  |  |  |

The model was adjusted for sex, age, smoking status, alcohol consumption, education level, marital status, hypertension, diabetes, cancer, lung disease, liver disease, eGFR, platelets, BUN, CRP, and SUA (excluding the stratification variable).

**Table S6. Associations of lipid-related indices with stage progression of CKM syndrome**

| **Variables** | **Model1** |  | **Model2** |  | **Model3** |  |
| --- | --- | --- | --- | --- | --- | --- |
|  | **OR (95%CI)** | **P value** | **OR (95%CI)** | **P value** | **OR (95%CI)** | **P value** |
| AIP | 0.32 (0.25, 0.39) | <0.001 | 0.30 (0.24, 0.37) | <0.001 | 0.39 (0.31, 0.50) | <0.001 |
| Non-HDL-C | 0.16 (0.09, 0.28) | <0.001 | 0.15 (0.08, 0.27) | <0.001 | 0.29 (0.16, 0.54) | <0.001 |
| NHHR | 0.22 (0.15, 0.31) | <0.001 | 0.19 (0.13, 0.28) | <0.001 | 0.31 (0.21, 0.46) | <0.001 |
| LCI | 0.41 (0.35, 0.49) | <0.001 | 0.40 (0.33, 0.48) | <0.001 | 0.51 (0.42, 0.62) | <0.001 |
| RC | 0.58 (0.49, 0.68) | <0.001 | 0.56 (0.48, 0.67) | <0.001 | 0.71 (0.60, 0.84) | <0.001 |
| LAP | 0.51 (0.44, 0.60) | <0.001 | 0.46 (0.39, 0.54) | <0.001 | 0.66 (0.55, 0.78) | <0.001 |
| VAI | 0.39 (0.32, 0.48) | <0.001 | 0.34 (0.28, 0.42) | <0.001 | 0.46 (0.37, 0.57) | <0.001 |
| TyG | 0.49 (0.44, 0.55) | <0.001 | 0.48 (0.43, 0.55) | <0.001 | 0.59 (0.52, 0.68) | <0.001 |
|  |  |  |  |  |  |  |

Model 1: unadjusted.

Model 2: adjusted for sex, age, smoking status, alcohol consumption, education level, and marital status.

Model 3: adjusted for Model 2 covariates plus hypertension, diabetes, cancer, lung disease, liver disease, eGFR, platelets, BUN, CRP, and SUA.

**Table S7. Associations between lipid-related indices and CKM syndrome**

| **Variables** | **Model1** |  | **Model2** |  | **Model3** |  |
| --- | --- | --- | --- | --- | --- | --- |
|  | **OR (95%CI)** | **P value** | **OR (95%CI)** | **P value** | **OR (95%CI)** | **P value** |
| AIP | 2.58 (2.12, 3.14) | <0.001 | 12.21 (9.15, 16.30) | <0.001 | 8.00 (5.64, 11.35) | <0.001 |
| Non-HDL-C | 11.37 (6.38, 20.27) | <0.001 | 630.68 (277.88, 1431.37) | <0.001 | 659.15 (242.81, 1789.39) | <0.001 |
| NHHR | 8.50 (5.93, 12.19) | <0.001 | 160.52 (94.31, 273.20) | <0.001 | 143.46 (74.92, 274.69) | <0.001 |
| LCI | 2.36 (1.99, 2.81) | <0.001 | 11.34 (8.73, 14.73) | <0.001 | 10.22 (7.41, 14.08) | <0.001 |
| RC | 1.69 (1.41, 2.02) | <0.001 | 3.65 (2.86, 4.66) | <0.001 | 2.02 (1.54, 2.64) | <0.001 |
| LAP | 1.23 (1.06, 1.44) | 0.008 | 7.49 (5.89, 9.52) | <0.001 | 4.05 (3.04, 5.38) | <0.001 |
| VAI | 1.28 (1.08, 1.53) | 0.005 | 10.81 (8.19, 14.25) | <0.001 | 6.74 (4.83, 9.42) | <0.001 |
| TyG | 1.81 (1.63, 2.00) | <0.001 | 4.34 (3.72, 5.07) | <0.001 | 2.59 (2.14, 3.12) | <0.001 |
|  |  |  |  |  |  |  |

Model 1: unadjusted.

Model 2: adjusted for sex, age, smoking status, alcohol consumption, education level, and marital status.

Model 3: adjusted for Model 2 covariates plus hypertension, diabetes, cancer, lung disease, liver disease, eGFR, platelets, BUN, CRP, and SUA.

**Table S8. Associations between lipid-related indices and CVD incidence in a population with CKM syndrome stages 0-3**

| **Variables** | **Model1** |  | **Model2** |  | **Model3** |  |
| --- | --- | --- | --- | --- | --- | --- |
|  | **HR (95%CI)** | **P value** | **HR (95%CI)** | **P value** | **HR (95%CI)** | **P value** |
| AIP | 1.38 (1.18, 1.60) | <0.001 | 1.39 (1.19, 1.63) | <0.001 | 1.23 (1.04, 1.45) | 0.014 |
| Non-HDL-C | 2.61 (1.66, 4.08) | <0.001 | 2.31 (1.47, 3.65) | <0.001 | 1.74 (1.09, 2.78) | 0.020 |
| NHHR | 2.03 (1.54, 2.67) | <0.001 | 1.95 (1.48, 2.58) | <0.001 | 1.60 (1.19, 2.14) | 0.002 |
| LCI | 1.44 (1.26, 1.65) | <0.001 | 1.41 (1.23, 1.62) | <0.001 | 1.26 (1.09, 1.46) | 0.001 |
| RC | 1.10 (0.97, 1.25) | 0.137 | 1.11 (0.98, 1.27) | 0.099 | 1.02 (0.91, 1.15) | 0.741 |
| LAP | 1.48 (1.30, 1.68) | <0.001 | 1.50 (1.31, 1.71) | <0.001 | 1.33 (1.15, 1.53) | <0.001 |
| VAI | 1.42 (1.23, 1.63) | <0.001 | 1.41 (1.21, 1.63) | <0.001 | 1.25 (1.07, 1.47) | 0.005 |
| TyG | 1.19 (1.10, 1.29) | <0.001 | 1.19 (1.10, 1.29) | <0.001 | 1.10 (1.01, 1.21) | 0.034 |
|  |  |  |  |  |  |  |

Model 1: unadjusted.

Model 2: adjusted for sex, age, smoking status, alcohol consumption, education level, and marital status.

Model 3: adjusted for Model 2 covariates plus hypertension, diabetes, cancer, lung disease, liver disease, eGFR, platelets, BUN, CRP, and SUA.

**Table S9. Associations of lipid-related indices with stage progression of CKM syndrome**

| **Variables** | **Model1** |  | **Model2** |  | **Model3** |  |
| --- | --- | --- | --- | --- | --- | --- |
|  | **OR (95%CI)** | **P value** | **OR (95%CI)** | **P value** | **OR (95%CI)** | **P value** |
| AIP | 0.31 (0.25, 0.38) | <0.001 | 1.49 (1.14, 1.95) | 0.004 | 1.69 (1.27, 2.24) | <0.001 |
| Non-HDL-C | 0.15 (0.08, 0.26) | <0.001 | 2.46 (1.22, 4.98) | 0.012 | 2.73 (1.32, 5.63) | 0.007 |
| NHHR | 0.21 (0.14, 0.29) | <0.001 | 2.56 (1.62, 4.05) | <0.001 | 3.01 (1.87, 4.84) | <0.001 |
| LCI | 0.41 (0.34, 0.48) | <0.001 | 1.49 (1.19, 1.88) | <0.001 | 1.64 (1.29, 2.08) | <0.001 |
| RC | 0.57 (0.48, 0.68) | <0.001 | 1.08 (0.92, 1.26) | 0.350 | 1.10 (0.93, 1.30) | 0.249 |
| LAP | 0.50 (0.43, 0.59) | <0.001 | 1.85 (1.48, 2.31) | <0.001 | 1.90 (1.51, 2.39) | <0.001 |
| VAI | 0.38 (0.32, 0.46) | <0.001 | 1.48 (1.15, 1.91) | 0.002 | 1.64 (1.26, 2.14) | <0.001 |
| TyG | 0.49 (0.44, 0.55) | <0.001 | 1.22 (1.06, 1.40) | 0.007 | 1.25 (1.07, 1.45) | 0.005 |
|  |  |  |  |  |  |  |

Model 1: unadjusted.

Model 2: adjusted for sex, age, smoking status, alcohol consumption, education level, marital status, and CKM.

Model 3: adjusted for Model 2 covariates plus hypertension, diabetes, cancer, lung disease, liver disease, eGFR, platelets, BUN, CRP, and SUA.


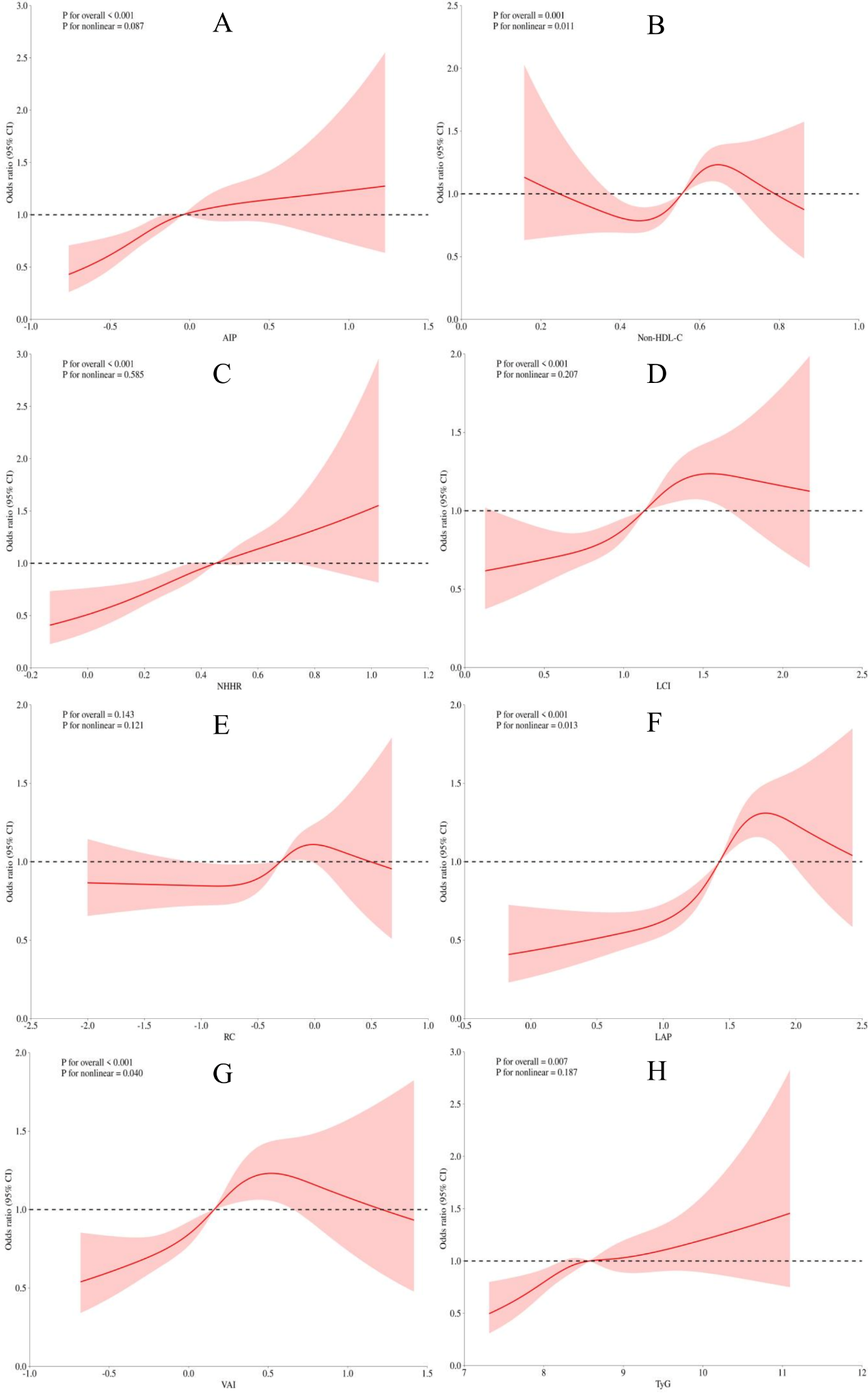


Figure S1.

Restricted cubic spline analysis of lipid-related indices (A: AIP; B: Non-HDL-C; C: NHHR; D: LCI; E: RC; F: LAP; G: VAI; H: TyG) in relation to the progression of CKM syndrome. Adjusted for sex, age, smoking status, alcohol consumption, education level, marital status, baseline CKM, hypertension, diabetes, cancer, lung disease, liver disease, eGFR, platelets, BUN, CRP, and SUA.
